# EvoProDom: evolutionary modeling of protein families by assessing translocations of protein domains

**DOI:** 10.1002/2211-5463.13245

**Published:** 2021-08-21

**Authors:** Gon Carmi, Alessandro Gorohovski, Milana Frenkel‐Morgenstern

**Affiliations:** ^1^ Cancer Genomics and BioComputing of Complex Diseases Lab The Azrieli Faculty of Medicine Bar‐Ilan University Safed Israel

**Keywords:** protein domains, protein evolution, translocations

## Abstract

Here, we introduce a novel ‘evolution of protein domains’ (EvoProDom) model for describing the evolution of proteins based on the ‘mix and merge’ of protein domains. We assembled and integrated genomic and proteomic data comprising protein domain content and orthologous proteins from 109 organisms. In EvoProDom, we characterized evolutionary events, particularly, translocations, as reciprocal exchanges of protein domains between orthologous proteins in different organisms. We showed that protein domains that translocate with highly frequency are generated by transcripts enriched in *trans*‐splicing events, that is, the generation of novel transcripts from the fusion of two distinct genes. In EvoProDom, we describe a general method to collate orthologous protein annotation from KEGG, and protein domain content from protein sequences using tools such as KoFamKOAL and Pfam. To summarize, EvoProDom presents a novel model for protein evolution based on the ‘mix and merge’ of protein domains rather than DNA‐based evolution models. This confers the advantage of considering chromosomal alterations as drivers of protein evolutionary events.

## Graphical abstract

The evolutionary model of protein domains builds new proteins. It is based on the ‘mix and merge’ of protein domains by means of chromosomal translocations, insertions, deletions, or duplication events in organisms during evolution. The changes have been identified in orthologous protein families using preserved, gained, and/or missing protein domains that appeared on evolutionary conserved chromosomal regions.

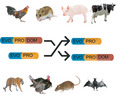

**Abbreviations**
DA: domain architecture
EvoProDom: evolution of protein domains
KEGG: Kyoto Encyclopedia of Genes and Genomes
KO: KEGG ortholog
PPI: protein–protein interaction

Proteins are composed from a set of domains that correspond to conserved regions with well‐defined functional and structural properties [1]. Consistent with the domain‐oriented view of proteins, domains cluster together to form domain architectures (DAs), that is, ordered sequences of domains. ‘Domain promiscuity’ or ‘domain mobility’ describes the diversity of DAs which participate in protein assembly. Analysis of domain promiscuity can reveal the mechanisms by which domains are gained or lost [2]. Marsh and Teichmann [1] described five mechanisms by which proteins gain domains: (a) gene fusion, namely, the fusion of a pair of adjacent genes via alternative splicing in noncoding intergenic regions; (b) exon extension, whereby exon regions expand into adjacent introns to encode a new domain; (c) exon recombination, involving the direct merging of two exons from two different genes; (d) intron recombination or exon shuffling, in which an exon inserts into an intron of a different gene; and (e) retroposition, where a sequence located within one gene is transposed into a different gene, along with a flanking genetic sequence. The properties of a gained domain, for example, position in protein sequence and number of exons, can identify which mechanism underlies domain addition. For example, gain of a multi‐exon domain at the C terminus is a result of gene fusion. Additionally, during metazoan evolution, new protein–protein interactions (PPIs) can emerge subsequent to the shuffling of exons encoding domains that mediate such interactions [3]. Work by Bornberg‐Bauer and Mar Albà [4] refined and expanded these mechanisms and introduced new concepts, such as intrinsically disordered regions, and implied links between the emergence of *de novo* domains and the appearance of *de novo* genes [4].

Here, we present a novel ‘evolution of protein domains (EvoProDom)’ model that determines the evolution of proteins, based on the ‘mix and merge’ approach of protein domains. In assembling this model, we collected and integrated genomic and proteomic data from 109 organisms. These data included protein domain and orthologous protein content. In EvoProDom, we accounted for evolutionary events, including translocations, namely, the reciprocal exchange of protein domains between orthologous proteins in different organisms. We found protein domains, which frequently appear in translocation events upon enrichment of *trans*‐splicing events, that is, when transcripts are producing upon slippage of two distinct genes [5]. EvoProDom, devised as a general method to obtain orthologous protein annotation and protein domain content, is based on predictions of these data from protein sequences using KoFamKOALA [6] and the Pfam search tool [7, 8]. The EvoProDom method can be implemented in other research fields such as proteomics [9], protein design [10] as well as assessing PPI in host‐virus systems [11].

## Materials and methods

The EvoProDom model is based on full genomic and annotated proteome data. In addition, the model utilizes orthologous protein annotation and protein domain content. Orthologous protein groups were used to group proteins (Refseqs) from different organisms, thereby linking protein domain changes among orthologous proteins with the corresponding groups of organisms. Orthologous proteins were realized as Kyoto Encyclopedia of Genes and Genomes (KEGG) orthologs (KOs) [12, 13]. Protein domain content was identified with Pfam domains, and this content was associated with proteins. Accordingly, orthologous proteins were considered as a group of proteins with the same KO number and proteins were considered as a group or list of Pfam domains. Both KO assignments and Pfam domains of proteins were predicted from protein sequences alone, using KoFamKOALA [6] and the Pfam search tool [7, 8], respectively. By utilizing these protein sequence‐based methods to attain protein domain content and orthologous protein annotation, new organisms are easily added to EvoProDom. Finally, statistical analysis was performed using r (R: A language and environment for statistical computing, 3.3.2, 2016).

### Data resources

The EvoProDom model was tested on a collection of 109 organisms of which 84 (77.06%), 6 (5.50%), and 19 (17.43%) are Eukaryota, Bacteria, and Viruses, respectively, with fully described genomes and annotated proteomes (Entrez/NCBI [14]) (Table 1). These organisms were grouped as follows: (a) 15 fish; (b) four subterranean, eight fossorial, and 21 aboveground animals [15, 16]; (c) 65 organisms with known PPIs (BioGrid version 3.5.173 [17, 18]); (d) 17 organisms with HiC datasets; (e) 4 cats; and (f) 15 pathogenic organisms [19]. Organisms with HiC datasets were obtained by searching for ‘HiC’ in the NCBI GEO database (Table 1). HiC is a NGS‐variant, high‐throughput method belonging to the chromosome conformation capture (3C) family. This method captures the 3D organization of a genome within the nucleolus by analysis of DNA contact frequencies, as estimated from HiC datasets [20].

**Table 1:** The EvoProDom model was applied to an assembly of organisms from diverse taxa belonging to superdomains, that is, Eukaryota, Viruses, and Bacteria. In total, 109 organisms were included in the ensemble and grouped as follows: (a) 15 fish; (b) four subterranean (S), eight fossorial (F), and 21 aboveground (A) animals (SFA) [15, 16]; (c) 65 organisms with known PPIs (BioGrid version 3.5.173, [17, 18]); (d) 17 organisms with HiC datasets (GEO_hic); (e) four cats; and (f) 15 pathogenic organisms [19]. Organisms with HiC datasets were obtained by searching for ‘HiC’ in the NCBI GEO database. Taxonomy ID, organism ID, organism name and common name are provided. Additionally, assembly and group classification are indicated. In addition, statistics for proteins and isoforms are included such that listed proteins are the longest isoforms and isoforms are alternative splicing variants. Total comprises both proteins and isoforms*. *Only proteins and isoforms with KO annotation are included. Organism ID is a 3–4 letter code, where the lowercase letter code corresponds to KEGG organisms and uppercase letters correspond to organisms not included in the KEGG database.

Organism ID	Organism name	Super kingdom	Ecology	Common name	Source^a^	Assembly	Total	Proteins	Isoforms
aga	*Anopheles gambiae* PEST	Eukaryota	na	African malaria mosquito	biogrid_3.5.173	GCF_000005575.2_AgamP3	6802	5928	874
aju	*Acinonyx jubatus*	Eukaryota	na	Cheetah	Cats	GCF_001443585.1_aciJub1	19 242	13 018	6224
ame	*Apis mellifera*	Eukaryota	na	honey bee	biogrid_3.5.173	GCF_000002195.4_Amel_4.5	12 559	6016	6543
ani	*Emericella nidulans* FGSC A4	Eukaryota	na	Aspergillus nidulans	biogrid_3.5.173	GCF_000149205.2_ASM14920v2	3925	3925	0
ASM	*Astyanax mexicanus*	Eukaryota	na	Mexican tetra	Fish	GCF_000372685.2_Astyanax_mexicanus‐2.0	29 294	16 593	12 701
ath	*Arabidopsis thaliana*	Eukaryota	na	Thale cress	GEO_hic, biogrid_3.5.173	GCF_000001735.4_TAIR10.1	21 347	11 664	9683
bsp	*Bacillus subtilis* PY79	Bacteria	na	na	GEO_hic	GCF_000497485.1_ASM49748v1	2399	2399	0
bsu	*Bacillus subtilis* 168	Bacteria	na	na	biogrid_3.5.173	GCF_002009135.1_ASM200913v1	2425	2425	0
bta	*Bos taurus*	Eukaryota	A	Cattle	biogrid_3.5.173,SFA	GCF_002263795.1_ARS‐UCD1.2	46 970	15 444	31 526
CAA	*Carassius auratus*	Eukaryota	na	Goldfish	Fish	GCF_003368295.1 ASM336829v1	66 282	34 815	31 467
cal	*Candida albicans* SC5314	Eukaryota	na	na	biogrid_3.5.173, Jones, *et al*. 2008	GCF_000182965.3_ASM18296v3	3419	3419	0
CAP	*Cavia porcellus*	Eukaryota	A	Domestic guinea pig	biogrid_3.5.173,SFA	GCF_000151735.1 Cavpor3.0	27 511	14 502	13 009
ccar	*Cyprinus carpio*	Eukaryota	na	Common carp	Fish	GCF_000951615.1_common_carp_genome	32 539	24 182	8357
ccr	*Caulobacter vibrioides*	Bacteria	na	na	GEO_hic	GCF_000006905.1_ASM690v1	1994	1994	0
cel	*Caenorhabditis elegans*	Eukaryota	na	Nematode	GEO_hic, biogrid_3.5.173	GCF_000002985.6_WBcel235	7918	5462	2456
cfa	*Canis familiaris*	Eukaryota	na	Dog	biogrid_3.5.173	GCF_000002285.3_CanFam3.1	41 761	14 307	27 454
cge	*Cricetulus griseus*	Eukaryota	A	Chinese hamster	biogrid_3.5.173, SFA	GCF_000419365.1_C_griseus_v1.0	23 914	14 931	8983
CHA	*Chrysochloris asiatica*	Eukaryota	S	Cape golden mole	SFA	GCF_000296735.1_ChrAsi1.0	19 180	14 764	4416
CHL	*Chinchilla lanigera*	Eukaryota	A	Long‐tailed chinchilla	SFA	GCF_000276665.1_ChiLan1.0	32 225	14 466	17 759
COC	*Condylura cristata*	Eukaryota	F	Star‐nosed mole	SFA	GCF_000260355.1_ConCri1.0	21 431	12 911	8520
COG	*Cottoperca gobio*	Eukaryota	na	Channel bull blenny	Fish	GCF_900634415.1 fCotGob3.1	27 249	15 024	12 225
cre	*Chlamydomonas reinhardtii*	Eukaryota	na	Green algae	biogrid_3.5.173	GCF_000002595.1_v3.0	3874	3835	39
csab	*Chlorocebus sabaeus*	Eukaryota	na	Green monkey	biogrid_3.5.173	GCF_000409795.2_Chlorocebus_sabeus_1.1	44 091	14 550	29 541
DAN	*Dasypus novemcinctus*	Eukaryota	F	Nine‐banded armadillo	SFA	GCF_000208655.1_Dasnov3.0	26 476	15 213	11 263
ddi	*Dictyostelium discoideum* AX4	Eukaryota	na	na	biogrid_3.5.173	GCF_000004695.1_dicty_2.7	4517	4508	9
DIO	*Dipodomys ordii*	Eukaryota	F	Ord's kangaroo rat	SFA	GCF_000151885.1_Dord_2.0	21 281	14 129	7152
dme	*Drosophila melanogaster*	Eukaryota	A	Fruit fly	SFA, GEO_hic, biogrid_3.5.173	GCF_000001215.4_Release_6_plus_ISO1_MT	15 749	6630	9119
dre	*Danio rerio*	Eukaryota	na	Zebrafish	Fish, biogrid_3.5.173, GEO_hic	GCF_000002035.6_GRCz11	37 274	17 375	19 899
ecb	*Equus caballus*	Eukaryota	na	Horse	biogrid_3.5.173	GCF_002863925.1_EquCab3.0	44 295	15 529	28 766
eco	*Escherichia coli* str. K‐12 substr. MG1655	Bacteria	na	na	biogrid_3.5.173	GCF_001566335.1_ASM156633v1	3194	3194	0
ECTE	*Echinops telfairi*	Eukaryota	A	Small Madagascar hedgehog	SFA	GCF_000313985.1 EchTel2.0	16 955	13 827	3128
ELE	*Elephantulus edwardii*	Eukaryota	A	Cape elephant shrew	SFA	GCF_000299155.1 EleEdw1.0	18 981	15 255	3726
ERE	*Erinaceus europaeus*	Eukaryota	A	Western European hedgehog	SFA	GCF_000296755.1_EriEur2.0	21 873	14 153	7720
fca	*Felis catus*	Eukaryota	A	Domestic cat	SFA, cats	GCF_000181335.3_Felis_catus_9.0	39 855	14 572	25 283
FUD	*Fukomys damarensis*	Eukaryota	S	Damara mole‐rat	SFA	GCF_000743615.1_DMR_v1.0	31 386	14 138	17 248
gga	*Gallus gallus*	Eukaryota	A	Chicken	biogrid_3.5.173, SFA,GEO_hic	GCF_000002315.5_GRCg6a	35 502	11 947	23 555
gmx	*Glycine max*	Eukaryota	na	Soybean	biogrid_3.5.173	GCF_000004515.4_Glycine_max_v2.0	32 653	21 054	11 599
HCV	Hepatitis C virus	Viruses	na	HCV	biogrid_3.5.173, Jones *et al*. 2008	GCF_000861845.1_ViralProj15432	1	1	0
hgl	*Heterocephalus glaber*	Eukaryota	S	Naked mole‐rat	SFA	GCF_000247695.1_HetGla_female_1.0	31 478	14 565	16 913
HHV1	Human Herpesvirus 1	Viruses	na	Herpes simplex virus type 1	biogrid_3.5.173, Jones *et al*. 2008	GCF_000859985.2_ViralProj15217	27	27	0
HHV2	Human Herpesvirus 2	Viruses	na	HHV2	biogrid_3.5.173	GCF_000858385.2_ViralProj15218	27	27	0
HHV3	Human Herpesvirus 3	Viruses	na	Varicella‐zoster virus	biogrid_3.5.173, Jones *et al*. 2008	GCF_000858285.1_ViralProj15198	6	6	0
HHV4	Human gammaherpesvirus 4	Viruses	na	EBV	GEO_hic, biogrid_3.5.173	GCF_002402265.1_Decoy	21	19	2
HHV5	Human Herpesvirus 5	Viruses	na	Human cytomegalovirus	biogrid_3.5.173, Jones *et al*. 2008	GCF_000845245.1_ViralProj14559	16	16	0
HHV6A	Human Herpesvirus 6A	Viruses	na	HHV6A	biogrid_3.5.173	GCF_000845685.1_ViralProj14462	5	5	0
HHV6B	Human Herpesvirus 6B	Viruses	na	HHV6B	biogrid_3.5.173	GCF_000846365.1_ViralProj14422	5	5	0
HHV7	Human Herpesvirus 7	Viruses	na	HHV7	biogrid_3.5.173, Jones *et al*. 2008	GCF_000848125.1_ViralProj14625	4	4	0
HHV8	Human gammaherpesvirus 8	Viruses	na	KSHV	GEO_hic, biogrid_3.5.173, Jones *et al*. 2008	GCF_000838265.1_ViralProj14158	8	8	0
HIV1	Human Immunodeficiency Virus 1	Viruses	na	HIV1	biogrid_3.5.173, Jones *et al*. 2008	GCF_000864765.1_ViralProj15476	5	5	0
HIV2	Human Immunodeficiency Virus 2	Viruses	na	HIV2	biogrid_3.5.173, Jones *et al*. 2008	GCF_000856385.1_ViralProj14991	5	5	0
HPV10	Human papillomavirus type 10	Viruses	na	HPV10	biogrid_3.5.173, Jones *et al*. 2008	GCF_000864905.1_ViralProj15504	7	6	1
HPV16	Human papillomavirus 16	Viruses	na	HPV16	biogrid_3.5.173, GEO_hic, Jones *et al*. 2008	GCF_000863945.3_ViralProj15505	7	7	0
HPV6b	Human papillomavirus type 6b	Viruses	na	HPV6b	biogrid_3.5.173, Jones *et al*. 2008	GCF_000861945.1_ViralProj15454	6	6	0
hsa	*Homo sapiens*	Eukaryota	A	Human	biogrid_3.5.173, SFA, GEO_hic	GCF_000001405.37_GRCh38.p11	76 306	14 484	61 822
ICT	*Ictidomys tridecemlineatus*	Eukaryota	F	Thirteen‐lined ground squirrel	SFA	GCF_000236235.1 SpeTri2.0	28 828	14 776	14 052
lav	*Loxodonta africana*	Eukaryota	A	African savanna elephant	SFA	GCF_000001905.1_Loxafr3.0	30 929	15 683	15 246
lcf	*Lates calcarifer*	Eukaryota	na	Barramundi perch	Fish	GCF_001640805.1_ASM164080v1	31 308	17 416	13 892
lcm	*Latimeria chalumnae*	Eukaryota	na	Coelacanth	Fish	GCF_000225785.1_LatCha1	22 318	13 088	9230
LEO	*Lepisosteus oculatus*	Eukaryota	na	Spotted gar	Fish	GCF_000242695.1 LepOcu1	28 773	12 422	16 351
MAM	*Macaca mulatta*	Eukaryota	na	Rhesus monkey	biogrid_3.5.173, GEO_hic	GCF_003339765.3 Mmul_10	49 563	15 063	34 500
MARM	*Marmota marmota*	Eukaryota	F	European marmot	SFA	GCF_001458135.1 marMar2.1	23 284	15 082	8202
mge	*Mycoplasma genitalium*	Bacteria	na	na	Jones *et al*. 2008	GCF_000027325.1_ASM2732v1	265	265	0
mgp	*Meleagris gallopavo*	Eukaryota	na	Turkey	biogrid_3.5.173	GCF_000146605.2_Turkey_5.0	20 631	11 045	9586
MIO	*Microtus ochrogaster*	Eukaryota	F	prairie vole	SFA	GCF_000317375.1_MicOch1.0	23 045	14 950	8095
mmu	*Mus musculus*	Eukaryota	A	House mouse	biogrid_3.5.173, SFA, GEO_hic	GCF_000001635.26_GRCm38.p6	54 095	15 939	38 156
mtv	*Mycobacterium tuberculosis* H37Rv	Bacteria	na	na	biogrid_3.5.173, Jones *et al*. 2008	GCF_000195955.2_ASM19595v2	1874	1874	0
ncc	*Notothenia coriiceps*	Eukaryota	na	Black rockcod	Fish	GCF_000735185.1_NC01	17 089	12 300	4789
ncr	*Neurospora crassa* OR74A	Eukaryota	na	na	biogrid_3.5.173	GCF_000182925.2_NC12	4303	3798	505
NEL	*Neotoma lepida*	Eukaryota	A	Desert woodrat	SFA	GCF_001675575.1 ASM167557v1	11 060	11 060	0
nfu	*Nothobranchius furzeri*	Eukaryota	na	Turquoise killifish	Fish	GCF_001465895.1_Nfu_20140520	25 760	15 051	10 709
ngi	*Nannospalax galili*	Eukaryota	S	Upper Galilee mountains blind mole rat	SFA	GCF_000622305.1_S.galili_v1.0	28 587	15 163	13 424
nle	*Nomascus leucogenys*	Eukaryota	na	Northern white‐cheeked gibbon	GEO_hic	GCF_000146795.2_Nleu_3.0	27 130	14 001	13 129
nto	*Nicotiana tomentosiformis*	Eukaryota	na	Tobacco	biogrid_3.5.173	GCF_000390325.2_Ntom_v01	21 031	12 501	8530
oaa	*Ornithorhynchus anatinus*	Eukaryota	A	platypus	SFA	GCF_000002275.2_Ornithorhynchus_anatinus_5.0.1	13 803	10 377	3426
oas	*Ovis aries*	Eukaryota	na	Sheep	biogrid_3.5.173	GCF_000298735.2_Oar_v4.0	31 319	14 663	16 656
OCD	*Octodon degus*	Eukaryota	F	Degu	SFA	GCF_000260255.1_OctDeg1.0	20 663	15 343	5320
ocu	*Oryctolagus cuniculus*	Eukaryota	na	Rabbit	biogrid_3.5.173, GEO_hic	GCF_000003625.3_OryCun2.0	27 567	14 450	13 117
ola	*Oryzias latipes*	Eukaryota	na	Japanese medaka	Fish	GCF_002234675.1_ASM223467v1	31 537	15 135	16 402
ORA	*Orycteropus afer afer*	Eukaryota	*A*	Aardvark	SFA	GCF_000298275.1_OryAfe1.0	19 243	14 511	4732
ORM	*Oryzias melastigma*	Eukaryota	na	Indian medaka	Fish	GCF_002922805.1 Om_v0.7.RACA	29 506	15 615	13 891
osa	*Oryza sativa Japonica*	Eukaryota	na	Rice	biogrid_3.5.173	GCF_001433935.1_IRGSP‐1.0	18 258	12 404	5854
PAP	*Panthera pardus*	Eukaryota	na	Leopard	Cats	GCF_001857705.1_PanPar1.0	42 102	14 693	27 409
PEF	*Perca flavescens*	Eukaryota	na	Yellow perch	Fish	GCF_004354835.1 PFLA_1.0	30 056	16 335	13 721
PEM	*Peromyscus maniculatus bairdii*	Eukaryota	A	Prairie deer mouse	SFA	GCF_000500345.1_Pman_1.0	33 249	15 592	17 657
pfa	*Plasmodium falciparum* 3D7	Eukaryota	na	Malaria parasite P. falciparum	biogrid_3.5.173, Jones *et al*. 2008	GCF_000002765.4_ASM276v2	2001	1973	28
phu	*Pediculus humanus corporis*	Eukaryota	na	Human body louse	biogrid_3.5.173	GCF_000006295.1_JCVI_LOUSE_1.0	5292	5290	2
pret	*Poecilia reticulata*	Eukaryota	na	Guppy	Fish	GCF_000633615.1_Guppy_female_1.0_MT	30 412	15 280	15 132
ptg	*Panthera tigris altaica*	Eukaryota	na	Tiger	Cats	GCF_000464555.1_PanTig1.0	21 205	13 229	7976
ptr	*Pan troglodytes*	Eukaryota	A	Chimpanzee	biogrid_3.5.173,SFA	GCF_002880755.1_Clint_PTRv2	57 743	14 939	42 804
rcu	*Ricinus communis*	Eukaryota	na	castor bean	biogrid_3.5.173	GCF_000151685.1_JCVI_RCG_1.1	14 121	10 018	4103
rno	*Rattus norvegicus*	Eukaryota	A	Norway rat	biogrid_3.5.173,SFA	GCF_000001895.5_Rnor_6.0	40 251	16 426	23 825
sasa	*Salmo salar*	Eukaryota	na	Atlantic salmon	Fish	GCF_000233375.1_ICSASG_v2	63 095	28 784	34 311
sce	*Saccharomyces cerevisiae* S288c	Eukaryota	na	Baker's yeast	biogrid_3.5.173,GEO_hic	GCF_000146045.2_R64	3588	3588	0
SIV	Simian Immunodeficiency Virus	Viruses	na	SIV	biogrid_3.5.173	GCF_000863925.1_ViralProj15501	4	4	0
sly	*Solanum lycopersicum*	Eukaryota	na	Tomato	biogrid_3.5.173	GCF_000188115.3_SL2.50	17 131	11 914	5217
smo	*Selaginella moellendorffii*	Eukaryota	na	na	biogrid_3.5.173	GCF_000143415.4_v1.0	19 207	14 135	5072
SOA	*Sorex araneus*	Eukaryota	A	European shrew	SFA	GCF_000181275.2 SorAra2.0	17 318	14 075	3243
sot	*Solanum tuberosum*	Eukaryota	na	Potato	biogrid_3.5.173	GCF_000226075.1_SolTub_3.0	17 431	12 698	4733
spo	*Schizosaccharomyces pombe*	Eukaryota	na	Fission yeast	GEO_hic, biogrid_3.5.173	GCF_000002945.1_ASM294v2	3053	3053	0
spu	*Strongylocentrotus purpuratus*	Eukaryota	na	Purple sea urchin	biogrid_3.5.173	GCF_000002235.4_Spur_4.2	12 330	8773	3557
ssc	*Sus scrofa*	Eukaryota	A	Pig	biogrid_3.5.173,SFA	GCF_000003025.6_Sscrofa11.1	47 355	15 297	32 058
SV40	Simian Virus 40	Viruses	na	Macaca mulatta polyomavirus 1	biogrid_3.5.173	GCF_000837645.1_ViralProj14024	1	1	0
TMV	Tobacco Mosaic Virus	Viruses	na	TMV	biogrid_3.5.173	GCF_000854365.1_ViralProj15071	0	0	0
URP	*Urocitellus parryii*	Eukaryota	F	Arctic ground squirrel	SFA	GCF_003426925.1 ASM342692v1	27 132	14 370	12 762
USM	*Ustilago maydis* 521	Eukaryota	na	na	biogrid_3.5.173	GCF_000328475.2 Umaydis521_2.0	3265	3257	8
VAV	*Vaccinia Virus*	Viruses	na	na	biogrid_3.5.173	GCF_000860085.1_ViralProj15241	24	24	0
vvi	*Vitis vinifera*	Eukaryota	na	Wine grape	biogrid_3.5.173	GCF_000003745.3_12X	20 127	12 120	8007
xla	*Xenopus laevis*	Eukaryota	na	African clawed frog	biogrid_3.5.173	GCF_001663975.1_Xenopus_laevis_v2	40 278	21 671	18 607
zma	*Zea mays*	Eukaryota	na	Maize	biogrid_3.5.173	GCF_000005005.2_B73_RefGen_v4	24 391	14 736	9655

^a^ Jones *et al*. 2008 [19].

### Orthologous protein annotation

Orthologous annotation was based on KEGG orthologs, or KO groups [12, 13]. Proteins were assigned to KO groups using KoFamKOALA, a Hidden Markov Model (HMM) profile‐based search tool [6]. To this end, an in‐house script was written to automatically assign proteins to KO groups using KoFamKOALA [6], based on protein sequence. Thus, only proteins with a unique KO annotation were collected. Additionally, an organism code was generated by selecting 3–4 letters from an organism's name in uppercase format (a lower case code represents organisms from the KEGG database; Table 1).

### Protein domain detection

Pfam (release 32.0, http://pfam.xfam.org/about) domains were predicted from protein sequences, using a Hidden Markov Model (HMM)‐based search tool [7, 8]. Accordingly, protein domain content was derived from protein sequences using an in‐house script. Additionally, each Pfam domain was classified, based on membership in super‐families (‘clan' as per pfam nomenclature). These data were added to the protein domain content of every protein.

### EvoProDomDB

Genomic and proteomic data, along with orthologous proteins and protein domain content data, were collated by shared data. The resulting relational database, EvoProDomDB, was written in MySQL on MariaDB (10.0.26, https://mariadb.org/about/) to generate an efficient search engine. The EvoProDom model was implemented and tested on the MySQL database (EvoProDomDB). EvoProDomDB was organized with orthologous proteins and protein content for the 2 190 207 protein products (1 123 544 full length and 1 066 663 isoforms) (Table 1), which are distributed among 23 147 KO groups, containing 17 929 unique Pfam domains.

**Fig. 1:**
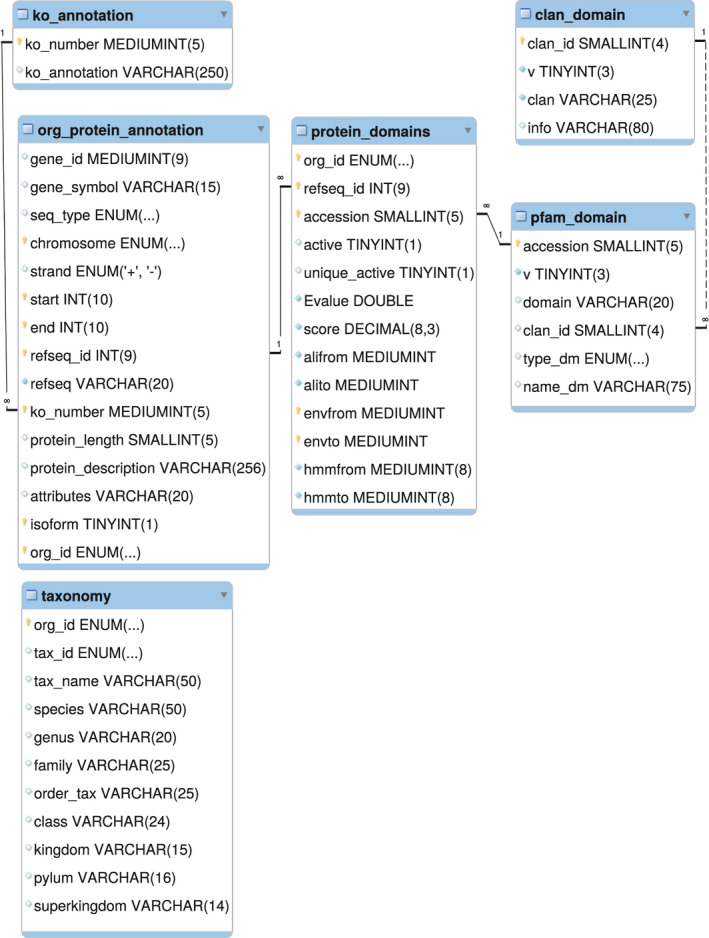
The MySQL scheme for EvoProDomDB. Six‐relation tables were included. Of these, four contained data regarding taxonomy (taxonomy), KO (ko_annotation,), super‐families (clan_domain), pfam domains (pfam_domain), such as taxonomy ranks, for example, genus and species, KO, domain and super‐family descriptions, respectively. The main relational tables contain protein, genomic and proteomic data (org_protein_annotation), as well as protein domain content (pfam data; see the main text for details).

The Pfam domains were distributed among 629 super‐families, while EvoProDomDB integrated data for 109 organisms from diverse taxa. EvoProDomDB was built from six relational tables sharing common features, for example, organism identity and other features (Fig. 1). Relational tables, taxonomy, ko_annotation, clan_domain, and pfam_domain provided the annotation data for taxonomy rankings, for example, genus and species, KO assignments, domain, and super‐family descriptions, respectively.

**Fig. 2:**
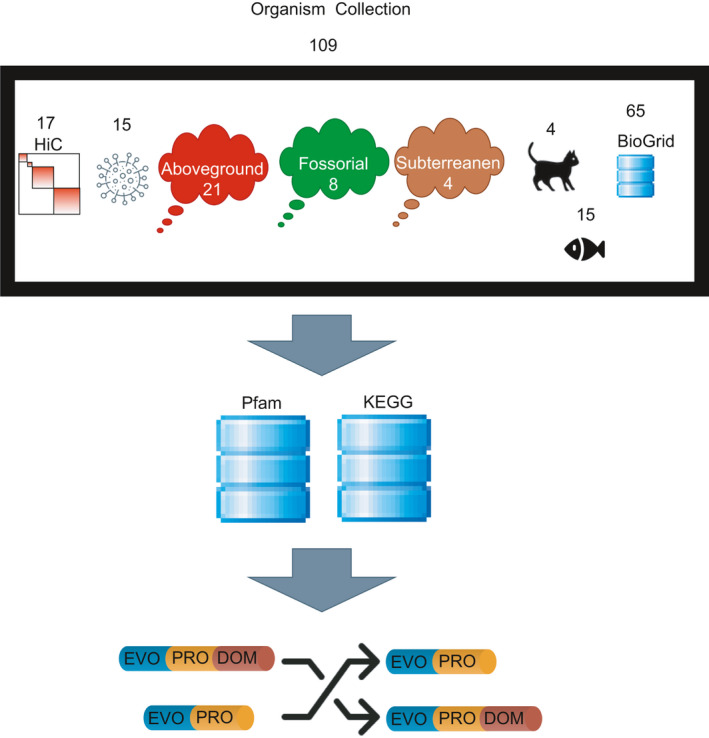
Study workflow: A collection of 109 organisms was used to implement and test the EvoProDom model. The collection included six categories: (a) 15 fish; (ii) four subterranean, eight fossorial and 21 aboveground animals [15, 16]; (c) 65 organisms with known PPIs (BioGrid version 3.5.173, [17, 18]); (d) 17 organisms with HiC datasets; (e) four cats; and (f) 15 pathogenic organisms [19]. Protein domains were predicted using the Pfam (release 32.0) database, along with the search tool [7, 8]. Orthologous proteins were defined as belonging to a KEGG [12, 13] ortholog (KO) group. Assignment to a KO group was obtained using KofamKOALA [6].

Protein genomic and proteomic data, along with protein domain content, were included in the relational tables as org_protein_annotation and Pfam data, respectively. Additionally, genomic and proteomic data were also included, for example, gene_symbol, chromosome, strand, refseq_id, protein length, and protein description. To these data, the KO number was added (ko_number). Proteomic and genomic data were uniquely linked by the longest isoform identification (isoform). Protein domain content was comprised from standard Pfam domains as retrieved from the Pfam search tool output [7, 8], and computed data that identified nonoverlapping Pfam domains with maximal score (putative) delimited by ‘envfrom’ and ‘envto’ coordinates. These coordinates delineate the largest region within the protein sequence in which a Pfam domain was predicted. Unique putative domain refers to the highest scoring domain among multiple copies of same putative domains. To collect these data, both standard and custom scripts were written and combined to form a pipeline that included construction of EvoProDomDB using in‐house bash and perl scripts. The EvoProDom model was implemented as Perl with MySQL queries to retrieve data from EvoProDomDB and bash scripts. These data sources and databases are summarized in the study workflow (Fig. 2).

## Results

### The EvoProDom model

We hypothesized that proteins evolve by means of ‘mix and merge’ or ‘shuffling’ of protein domains, which correspond to distinct functional units [1, 21, 22]. The evolutionary model that describes protein evolution as a function of protein domain dynamics was termed EvoProDom. The EvoProDom model defines and formulates standard evolutionary mechanisms, such as translocations, duplications, and indel (insertion and deletion) events, which acted upon protein domains that are recognized as Pfam domains [7, 8]. According to the EvoProDom model, proteins gained or lost function due to the respective presence or absence of function‐conferring domains. Accordingly, proteins were modeled as sets of protein domains and evolutionary events, such as translocations, were defined. These describe the gain and loss of particular domains among domain sets or DAs. The KEGG database catalogs diverse taxa and creates groups of orthologous proteins (KOs) based on shared function. Thus, all members of a KO group are orthologous proteins [6, 12, 13]. In the EvoProDom model, proteins were assigned to KO groups (see Materials and methods). Consequently, translocation events were mapped to groups of organisms according to underlying changes in DAs. Thus, evolutionary events, which acted upon domains and are manifested as changes in DAs, are reflected at the organism level. A link between changes at these two levels was, therefore, established. The EvoProDom model was implemented with and tested on the EvoProDomDB (see Materials and methods). In total, 6286 translocation events, involving 94 protein super‐families, were found (Table 2, Tables S1 and S2). This result indicates the existence of multiple evolutionary translocation events, as defined by the model.

**Table 2:** Translocation events per superfamily (counts). Translocations are characterized by mobile domains in organisms classified based on superdomain taxonomy*. These organism groups are assigned representative superdomain taxonomy if all organisms share same superdomain taxonomy. Otherwise, they are assigned as ‘Mixed’. Finally, translocations are classified based on organism group classification to superdomains, for example, Eukaryota‐Eukaryota, which represent the majority of translocations (over 99%) (Translocation Class). The most frequent clan for Eukaryota‐Eukaryota is Ig. Related to Tables S1 and S2. *Superdomain taxa are Eukaryota, Viruses, and Bacteria. Super‐family annotation is provided (Super family Description).

Translocation class	Super family Id	Super family name	Counts	Super family description
Eukaryota‐Eukaryota	0011.26	Ig	1144	Immunoglobulin superfamily
Eukaryota‐Eukaryota	0010.21	SH3	630	Src homology‐3 domain
Eukaryota‐Eukaryota	0465.3	Ank	529	Ankyrin repeat superfamily
Eukaryota‐Eukaryota	0001.27	EGF	414	EGF superfamily
Eukaryota‐Eukaryota	0361.4	C2H2‐zf	390	Classical C2H2 and C2HC zinc fingers
Eukaryota‐Eukaryota	0022.32	LRR	282	Leucine Rich Repeat
Eukaryota‐Eukaryota	0020.25	TPR	246	Tetratrico peptide repeat superfamily
Eukaryota‐Eukaryota	0229.11	RING	242	Ring‐finger/U‐box superfamily
Eukaryota‐Eukaryota	0186.14	Beta_propeller	222	Beta propeller clan
Eukaryota‐Eukaryota	0221.11	RRM	210	RRM‐like clan
Eukaryota‐Eukaryota	9999.0	Unknown	208	null
Eukaryota‐Eukaryota	0159.16	E‐set	187	Ig‐like fold superfamily (E‐set)
Eukaryota‐Eukaryota	0466.3	PDZ‐like	165	PDZ domain‐like peptide‐binding superfamily
Eukaryota‐Eukaryota	0016.22	PKinase	164	Protein kinase superfamily
Eukaryota‐Eukaryota	0266.9	PH	141	PH domain‐like superfamily
Eukaryota‐Eukaryota	0023.34	P‐loop_NTPase	121	P‐loop containing nucleoside triphosphate hydrolase superfamily
Eukaryota‐Eukaryota	0220.12	EF_hand	115	EF‐hand like superfamily
Eukaryota‐Eukaryota	0511.3	Retroviral_zf	95	Retrovirus zinc finger‐like domains
Eukaryota‐Eukaryota	0271.7	F‐box	79	F‐box‐like domain
Eukaryota‐Eukaryota	0003.21	SAM	74	Sterile Alpha Motif (SAM) domain
Eukaryota‐Eukaryota	0390.4	zf‐FYVE‐PHD	47	FYVE/PHD zinc finger superfamily
Eukaryota‐Eukaryota	0357.4	SMAD‐FHA	37	SMAD/FHA domain superfamily
Eukaryota‐Eukaryota	0063.25	NADP_Rossmann	37	FAD/NAD(P)‐binding Rossmann fold Superfamily
Eukaryota‐Eukaryota	0123.18	HTH	34	Helix‐turn‐helix clan
Eukaryota‐Eukaryota	0680.1	WW	34	WW domain
Eukaryota‐Eukaryota	0167.15	Zn_Beta_Ribbon	33	Zinc beta‐ribbon
Eukaryota‐Eukaryota	0006.20	C1	25	Protein kinase C, C1 domain
Eukaryota‐Eukaryota	0306.4	HeH	24	LEM/SAP HeH motif
Eukaryota‐Eukaryota	0214.13	UBA	24	UBA superfamily
Eukaryota‐Eukaryota	0459.3	BRCT‐like	23	BRCT like
Eukaryota‐Eukaryota	0188.10	CH	23	Calponin homology domain
Eukaryota‐Eukaryota	0537.2	CCCH_zf	22	CCCH‐zinc finger
Eukaryota‐Eukaryota	0004.20	Concanavalin	20	Concanavalin‐like lectin/glucanase superfamily
Eukaryota‐Eukaryota	0072.20	Ubiquitin	19	Ubiquitin superfamily
Eukaryota‐Eukaryota	0033.14	POZ	17	POZ domain superfamily
Eukaryota‐Eukaryota	0154.11	C2	11	C2 superfamily
Eukaryota‐Eukaryota	0007.18	KH	9	K‐Homology (KH) domain Superfamily
Eukaryota‐Eukaryota	0392.4	Chaperone‐J	8	Chaperone J‐domain superfamily
Eukaryota‐Eukaryota	0164.13	CUB	8	CUB clan
Eukaryota‐Eukaryota	0029.20	Cupin	8	Cupin fold
Eukaryota‐Eukaryota	0049.15	Tudor	8	Tudor domain 'Royal family'
Eukaryota‐Eukaryota	0172.17	Thioredoxin	8	Thioredoxin‐like
Eukaryota‐Eukaryota	0212.9	SNARE	8	SNARE‐like superfamily
Eukaryota‐Eukaryota	0124.15	Peptidase_PA	7	Peptidase clan PA
Eukaryota‐Eukaryota	0575.2	EFTPs	7	Translation proteins of Elongation Factors superfamily
Eukaryota‐Eukaryota	0137.15	HAD	7	HAD superfamily
Eukaryota‐Eukaryota	0021.18	OB	7	OB fold
Eukaryota‐Eukaryota	0364.4	Leu‐IlvD	7	LeuD/IlvD‐like
Eukaryota‐Eukaryota	0541.2	SH2‐like	6	SH2, phosphotyrosine‐recognition domain superfamily
Eukaryota‐Eukaryota	0671.1	AAA_lid	5	AAA+ ATPase lid domain superfamily
Eukaryota‐Eukaryota	0244.9	PGBD	5	PGBD superfamily
Eukaryota‐Eukaryota	0192.13	GPCR_A	5	Family A G protein‐coupled receptor‐like superfamily
Eukaryota‐Eukaryota	0173.11	STIR	5	STIR superfamily
Eukaryota‐Eukaryota	0602.2	Kringle	5	Kringle/FnII superfamily
Eukaryota‐Eukaryota	0642.1	SOCS_box	4	SOCS‐box like superfamily
Eukaryota‐Eukaryota	0178.16	PUA	4	PUA/ASCH superfamily
Eukaryota‐Eukaryota	0041.13	Death	4	Death Domain Superfamily
Eukaryota‐Eukaryota	0183.14	PAS_Fold	4	PAS domain clan
Eukaryota‐Eukaryota	0084.13	ADP‐ribosyl	3	ADP‐ribosylation Superfamily
Eukaryota‐Eukaryota	0015.20	MFS	3	Major Facilitator Superfamily
Eukaryota‐Eukaryota	0198.16	HHH	3	Helix‐hairpin‐helix superfamily
Eukaryota‐Eukaryota	0661.1	Gain	3	GPCR autoproteolysis inducing
Eukaryota‐Eukaryota	0497.3	GST_C	3	Glutathione S‐transferase, C‐terminal domain
Eukaryota‐Eukaryota	0030.16	Ion_channel	3	Ion channel (VIC) superfamily
Eukaryota‐Eukaryota	0107.12	KOW	2	KOW domain
Eukaryota‐Eukaryota	0492.3	S4	2	S4 domain superfamily
Eukaryota‐Eukaryota	0055.13	AMP‐binding_C	2	AMP‐binding enzyme C‐terminal domain superfamily
Eukaryota‐Eukaryota	0055.13	Nucleoplasmin	2	Nucleoplasmin‐like/VP (viral coat and capsid proteins) superfamily
Eukaryota‐Eukaryota	0027.15	RdRP	2	RNA‐dependent RNA polymerase
Eukaryota‐Eukaryota	0202.11	GBD	2	Galactose‐binding domain‐like superfamily
Eukaryota‐Eukaryota	0028.22	AB_hydrolase	2	Alpha/Beta hydrolase fold
Eukaryota‐Eukaryota	0677.1	GHMP_C	1	GHMP C‐terminal domain superfamily
Eukaryota‐Eukaryota	0025.14	His_Kinase_A	1	His Kinase A (phospho‐acceptor) domain
Eukaryota‐Eukaryota	0088.16	Alk_phosphatase	1	Alkaline phosphatase‐like
Eukaryota‐Eukaryota	0607.2	TNF_receptor	1	TNF receptor‐like superfamily
Mixed‐Mixed	0070.13	ACT	1	ACT‐like domain
Eukaryota‐Eukaryota	0113.13	GT‐B	1	Glycosyl transferase clan GT‐B
Eukaryota‐Eukaryota	0449.3	G‐PATCH	1	DExH‐box splicing factor binding site
Eukaryota‐Eukaryota	0144.13	Periplas_BP	1	Periplasmic binding protein like
Eukaryota‐Eukaryota	0505.3	Pentapeptide	1	Pentapeptide repeat
Eukaryota‐Eukaryota	0547.2	GF_recep_C‐rich	1	Growth factor receptor Cys‐rich
Eukaryota‐Mixed	0021.18	OB	1	OB fold
Eukaryota‐Eukaryota	0026.20	CU_oxidase	1	Multicopper oxidase‐like domain
Eukaryota‐Eukaryota	0110.12	GT‐A	1	Glycosyl transferase clan GT‐A
Eukaryota‐Eukaryota	0236.17	PDDEXK	1	PD‐(D/E)XK nuclease superfamily
Eukaryota‐Eukaryota	0672.1	p35	1	Baculovirus p35 protein superfamily
Eukaryota‐Eukaryota	0125.15	Peptidase_CA	1	Peptidase clan CA
Eukaryota‐Eukaryota	0117.11	uPAR_Ly6_toxin	1	uPAR/Ly6/CD59/snake toxin‐receptor superfamily
Eukaryota‐Eukaryota	0005.27	Kazal	1	Kazal like domain
Eukaryota‐Bacteria	9999.0	Unknown	1	null
Eukaryota‐Mixed	9999.0	Unknown	1	null
Eukaryota‐Eukaryota	0196.12	DSRM	1	DSRM‐like clan
Eukaryota‐Eukaryota	0381.4	Metallo‐HOrase	1	Metallo‐hydrolase/oxidoreductase superfamily
Eukaryota‐Eukaryota	0114.12	HMG‐box	1	HMG‐box like superfamily
Eukaryota‐Eukaryota	0109.12	CDA	1	Cytidine deaminase‐like (CDA) superfamily
Eukaryota‐Eukaryota	0552.2	Hect	1	Hect, E3 ligase catalytic domain
Eukaryota‐Eukaryota	0426.4	HRDC‐like	1	HRDC‐like superfamily
Eukaryota‐Eukaryota	0630.1	PSI	1	Plexin fold superfamily

### Mapping of genes to proteins and alternative splicing

EvoProDom combines genomic information (genes) with proteins, and in turn, proteins with Pfam domain composition. In addition, proteins assigned to KO groups were also included [6, 12, 13]. Genes may map to more than one mRNA transcript and, in turn, to more than one protein product, recognized by their Refseq id. These transcripts encode isoforms of a gene product and result from alternative splicing, that is, the inclusion of gene exons. Since protein domains mostly coincide with exons [1, 3, 5, 21], changes in protein domain content can account for changes in DAs as a result of translocation events. Therefore, to avoid confounding effects of alternative splicing, only the longest isoform was used in the model (see Materials and methods). As such, each gene was associated with a single protein product.

### Protein domain content

Overlapping domains within a protein are inconsistent with the linear structure of that protein. To resolve this issue for each overlapping group of domains, the highest scoring domain (the putative domain) was chosen. However, this procedure does remove multiple copies of putative domains. Translocation events require a unique set of nonoverlapping putative domains. To this end, a similar procedure was applied to remove multiple copies of putative domains by choosing domains with maximal score, subsequently referred to as unique putative domains.

### The DA as a basic unit in EvoProDom

According to the EvoProDom model, evolutionary events, such as translocations and indels, operated on protein domains and the organisms involved in orthologous groups, that is, KO and DAs. Therefore, EvoProDomDB enables organizing these data according to DA. Briefly, each orthologous group (KO) was partitioned into distinct sets (items), that is, a list of domains (DAs), and corresponding lists of proteins and organisms. Notably, duplicated organisms within these matched lists represent paralogous proteins. For each DA, gained and missing domains were determined from all DAs within a particular KO. Mobile and translocation domains, that is, domains that had undergone all translocation events, were determined from these data. In total, we found 6286 translocation events, involving 94 protein super‐families (Table 2, Tables S1 and S2). We identified 2042 mobile domains, 260 which had undergone translocation and 1782 that were involved in indel events (Tables S1 and S3).

### Evolutionary mechanisms represented in EvoProDom

#### Implementation of DAs

First, DAs were generated from EvoProDomDB, while filtering for putative and unique putative domains (see Materials and methods). DAs were uniquely identified as a (ko,item) pair. Each DA included: (a) a ko:item; (b) a Pfam domain list; (c) a list of organisms (org_id); (d) a list of refseq_ids; (e) a list of missing domains; and (f) a list of gained domains. Importantly, the list of organisms (c) and the list of refseq_ids (d) were matched lists, that is, the first refseq belonged to the first organism and the second refseq belonged to the second organism, etc. All other DA information was shared by all organisms and corresponding refseqs; namely, all refseqs were members of the same KO group and presented similar domain content (item). Gained and lost patterns [(e) and (f), above] were computed for each KO group across all DAs as items. Of note, the minimal number of DAs, that is, items, was two.

Domain architecture, the putative domain, and unique putative domain were formally defined as follows:

Definition: DA
  Algorithm: Let $p_{1},\;p_{2},\cdots ,\;p_{n\;}\subseteq D$, where $D\;=\;\{d_{1},d_{2},\cdots ,d_{m}\}$, is a set of protein domains and $p_{i}$is DA. Grouping of DAs into distinct groups is a partition of $p_{1},\;p_{2},\cdots ,\;p_{n}$.
Definition: Putative domains and unique putative domains
  Assumptions: Protein, $p=\;\left\{d_{1},d_{2},\cdots d_{m}\right\}$, must be DA, $c\left(d\right)\;\in R$ must be a score
  Algorithm: Domain d∈p is a putative domain if c(d) is maximal among overlapping or nested domains. A unique putative domain is the highest scoring putative domain among multiple copies of the same domain within p.

#### Translocation and indel events of a mobile domain

Informally, translocations of mobile domains involve gain/loss from/to orthologous proteins from two KO groups, in which mobile domains were determined by gain/loss patterns within a single KO group. Therefore, a mobile domain was described and formally defined. The main objective of the EvoProDom model was to reflect changes in domain content, namely, at the protein level, with the organism level. This highlights groups of organisms with orthologous proteins that share similar patterns of protein domain gain/loss. Protein domain composition was coupled with organisms by defining mobile and translocation domains. This was based on groups of organisms and their sizes, with orthologous proteins sharing the same protein domain composition. Protein domains were contained within orthologous proteins, or the domain missing from a protein, which was based on a number of organisms in each group, that is, orthologous proteins with and without a particular domain.

A mobile domain was defined as follows:

Assumptions: Let A,B,Tbe sets of organisms with proteins in a KO group, k, such that $=\;A\cup B,\;A\cap B=\emptyset ,\;O\in A\left\{p\in O\right|d_{x}\in p\},O\in B\left\{p\in O\right|d_{x}\notin p\}.$Organisms, O, in Acontain domain *d_x_* whereas organisms, O, in Black domain *d_x_*.
Algorithm: Unique putative domain $d_{x}$is mobile between organisms in A and in B if 4≤|A|<|T|‐4.

**Fig. 3:**
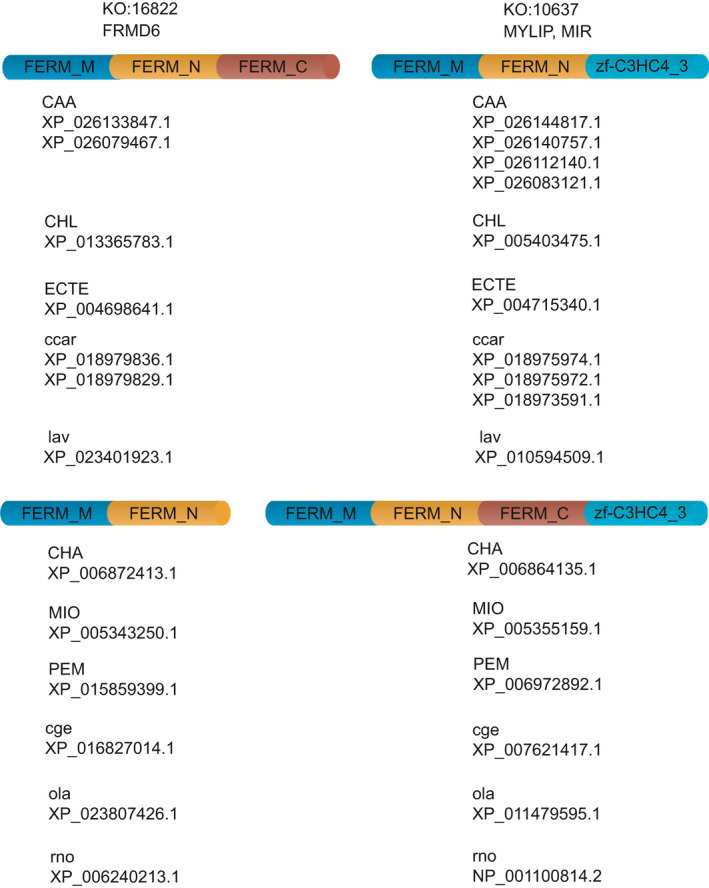
Illustration of translocation event for FERM_C. FERM_C (red domain) underwent a reciprocal translocation event between two orthologous protein groups 16822 (FRMD6) and 10637 (MYLIP, MIR). Accordingly, the red domain (FERM_C) is present in FRMD6 and absent from MYLIP for organisms CAA, etc., while for organisms CHA, etc., FERM_C is present in MYLIP and missing from FRMD6. FERM_C (FERM C‐terminal PH‐like domain); FERM. Orthologous proteins are indicated by refseqs for each organism, and multiple proteins per organism represent paralogue proteins. Organism codes are indicated in Table 1.

Next, translocations and indel events of mobile domains were described. Translocations and indel events are mutually exclusive events. Translocation domains comprise a subset of mobile domains showing patterns of gain and loss between two KO groups in a reciprocal manner, namely, a mobile domain that was gained and lost in the first and second orthologous group, and vice versa (Fig. 3). Similar to the definition of a mobile domain, translocation event criteria were defined for groups of organisms with four or more members. For example, a translocation event of the Pfam domain FERM_C (FERM C‐terminal PH‐like domain) in FERM (F for 4.1 protein, E for ezrin, R for radixin, and M for moesin) is shown in Fig. 3. In this translocation event, FERM_C was present in KEGG orthologous group number 16822, corresponding to FERM domain‐containing protein 6 (FRMD6). FERM_C was absent from the orthologous protein group number 10637, which corresponds to E3 ubiquitin‐protein ligase MYLIP [EC:2.3.2.27] (MYLIP, MIR) [23, 24, 25, 26]. This gain and loss pattern of FERM_C was observed among 29 orthologous proteins in two groups of organisms (A* and B*) consisting of five and six members, respectively. The first group, A*, which includes CAA (*Carassius auratus*, goldfish), CHL *Chinchilla lanigera*, long‐tailed chinchilla), ECTE (*Echinops telfairi*, small Madagascar hedgehog), ccar (*Cyprinus carpio*, common carp), and lav (*Loxodonta africana,* African savanna elephant), each contains at least one protein which gained and lost domain FERM_C in FRMD6 and MYLIP, respectively. The second group, B*, which includes CHA ( *asiatica*, Cape golden mole), MIO (*Microtus ochrogaster*, prairie vole), PEM (*Peromyscus maniculatus bairdii*, prairie deer mouse), cge (*Cricetulus griseus*, Chinese hamster), ola (*Oryzias latipes*, Japanese medaka), and rno (*Rattus norvegicus*, Norway rat), each contains at least one protein which gained and lost domain FERM_C in MYLIP and FRMD6, respectively (Table 1, Fig. 3). Since domain FERM_C showed reciprocal gain and loss patterns for a minimum of four organisms in, A* and B*, it was determined that this domain had undergone a translocation event and was referred to as a translocation domain (Fig. 3). Orthologous proteins are indicated by refseqs for each organism, with multiple proteins per organism representing paralogous proteins (Fig. 3).

Translocations and indel events were formally defined as follows:

Assumptions: Let $d_{x}$ be a mobile domain between $A_{i}$ and $B_{i}$ in $k_{i}$, where i=1,2, $A_{i},B_{i}$ are sets of organisms and $k_{i}$ are KO groups. Let $A^{*}=A_{1}\cap B_{2}$ and $B^{*}=A_{2}\cap B_{1}$.
Algorithm: Mobile domain $d_{x}$ undergoes translocation if $\left|A^{*}\right|,\;\left|B^{*}\right|\geq 4$. Otherwise, an indel event has occurred.

Over 77% of organisms in the EvoProDom database are eukaryotes. Therefore, translocation events are expected to predominately involve eukaryotes. To test this prediction, translocation events, which involve two organism groups (A^*^, B^*^), were classified based on superdomain taxonomy, namely, Eukaryota, Viruses, and Bacteria. Briefly, each organism group was assigned to the superdomain taxonomy shared by all organisms; otherwise, the group was assigned as ‘Mixed’. In these superdomain taxonomy assignments of organism groups, translocations were classified based on superdomain taxonomy, represented as composites of individual organism group assignments (A^*^–B^*^; Table S1). For example, Eukaryota‐Eukaryota consists of 6282 (99.94%) translocations (Tables S1 and S2). For this group, Ig_3 is the most frequent translocating domain (528/6282, 8.40%) and Ig is the most abundant superfamily (clan) (1144/6, 282, 18.21%; Table 2, Table S1). These results validate the prediction of overrepresentation of translocations involving only eukaryotes as a consequence of eukaryotes predominating in the EvoProDom database. Interestingly, a single translocation was assigned to the Eukaryota‐Bacteria group, which involved the FDX‐ACB domain. At the same time, three translocations were assigned to the Mixed group, that is, translocations involving at least one bacterial species in either organism group (Tables S1 and S2). This domain, ferredoxin‐fold anticodon binding (FDX‐ACB), is contained in Phenylalanine‐tRNA synthetase (PheRS, also known as Phenylalanine‐tRNA ligase) and is shared by bacteria and mitochondria [27, 28, 29, 30, 31, 32]. This translocation involves orthologous protein groups 01889, FARSA, pheS; phenylalanyl‐tRNA synthetase alpha chain [EC:6.1.1.20] and 01890, FARSB, pheT; phenylalanyl‐tRNA synthetase beta chain [EC:6.1.1.20] (Tables S1 and S2). These results indicate that translocations are not restricted to eukaryotes and support the theory of a bacterial origin of mitochondria. Moreover, examination of domains and protein orthologous groups (KO) revealed that they are common to bacteria species, for example, translocation domain Abhydrolase_1, which involves orthologous protein group (13700, ABHD6; abhydrolase domain‐containing protein 6 [EC:3.1.1.23]) was found in Alphaproteobacteria (e.g., ster Sphingopyxis terrae, tax_id33052), Betaproteobacteria (rhg Rhodoferax sediminis Gr‐4, tax_id2509614), Gammaproteobacteria (pfo Pseudomonas fluorescens Pf0–1, tax_id294), and Deltaproteobacteria (sur Stigmatella aurantiaca, tax_id41). The second orthologous protein group is 13703, ABHD11; abhydrolase domain‐containing protein 11 found in Alphaproteobacteria (e.g., abg Asaia bogorensis, tax_id91915) and Verrucomicrobia (e.g., mkc Methylacidiphilum kamchatkense, tax_id431057; Tables S1 and S2). These results point to possible translocations among bacteria, which share orthologous proteins with eukaryotes.

Similar to translocation events, the vast majority (96.67%) of indel events involve only eukaryotes (Table S3). The most frequent domain for indel class Eukaryota‐Eukaryota is SNF2_N, which belong to P‐loop_NTPase superfamily, with 290 indel events (Table S4) and ‘Unknown’ with 8382 indels (Table S5). However, we found 570 indel events which involve bacteria, 70 of which involve either domain gain in bacteria yet absence of the gene in eukaryotes or vice versa (Table S3). Interestingly, we found two collections of indel events involving two orthologous proteins, 01889, phenylalanyl‐tRNA synthetase alpha chain [EC:6.1.1.20] and 01890, phenylalanyl‐tRNA synthetase beta chain [EC:6.1.1.20]. For example, the collection of indel events for alpha chains, which contain PheRS_DBD1, PheRS_DBD2, and PheRS_DBD3 domains, is gained in eukaryotes; that is, the events are classified as Bacteria‐Eukaryota, Eukaryota‐Eukaryota, and Mixed‐Eukaryota. However, the Phe_tRNA‐synt_N domain is gained Bacteria, namely, indel events which are classified Mixed‐Bacteria and Eukaryota‐Bacteria (Table S3). These results show that indel events are not restricted to eukaryotes.

#### Duplication of domains

Unique putative Pfam domains form the basis for defining mobile and translocation events. For duplication events, putative domains were considered so as to retain nonoverlapping duplicates of Pfam domains (see Materials and methods). These putative domains were calculated for each orthologous protein group, that is, KO group, to assign duplicate status. This status varied among KO groups, and corresponded to ‘duplicated’ or ‘nonduplicated’ for a particular KO group and thus varied among KO groups. Therefore, the final duplication status of a Pfam domain was determined by the majority of duplicate status assignments for individual KO groups. For example, the final duplication status of a Pfam domain was ‘duplicated’ if the difference between the number of KOs with ‘duplicated’ to ‘nonduplicated’ was significant, namely, in the 99% percentile of the cumulative sum of the differences. Similarly, a final ‘nonduplicated’ status was determined when considering ‘nonduplicated’ to ‘duplicated’ differences. The duplicate status of a domain in a given KO group was determined based on consistency of domain copy number across all Das; that is, if constant across all DAs, then ‘nonduplicated’ was assigned. Otherwise, ‘duplicated’ was assigned.

Duplication was formally defined as follows:

Assumptions: Let $d_{x}$ be a putative domain, kobe the KO group with $da_{1},\;da_{2},\;\cdots .\;,\;da_{m}$ DAs of putative domains. Then, $d_{x}$is ‘nonduplicated’ in, ko if the copy number of $d_{x}$ is the same in each, otherwise $d_{x}$ is ‘duplicated’.
Algorithm: $d_{x}$ is duplicated if the difference between the number of KO groups where $d_{x}$ is ‘duplicated’ and the number of KO groups where it is ‘nonduplicated’ is significant (above 99% of the cumulative sum of the differences). A nonduplicated domain is similarly defined.

#### Translocation domains are enriched in chimeric transcripts

Chimeric transcripts are combined transcripts derived from two genes. Frenkel‐Morgenstern and Valencia [5] analyzed domain content enrichment within chimeric transcripts and found enriched domains belonging to the following super‐families (super‐family name): ANK (Ank), EFh (EF_hand), EGF‐like (EFG), GTP_EFTU (P‐loop_NTPase), IG‐like (E‐set), LRR (LRR), PH (zf‐FYVE‐PHD), Pkinase (PKinase), RING (RING), RRM (RRM), SH2 (SH2‐like), SH3 (SH3), WD40 (Beta_propeller), and ZnF (C2H2‐zf) [5]. Of these, EFh (EF_hand), EGF‐like (EFG), GTP_EFTU (P‐loop_NTPase), IG‐like(E‐set), Pkinase (PKinase), RRM (RRM), SH2 (SH2‐like), SH3 (SH3), WD40 (Beta_propeller), and ZnF (C2H2‐zf), findings confirmed by RNA‐seq data analysis [5]. These domains were found in high copy numbers within proteins, such as Ank [33, 34, 35] and WD40 [36], or as repeats or highly abundant within proteins, such as SH3 [37, 38]. Therefore, we hypothesized that highly abundant domains might have experienced a high number of translocation events. Therefore, we applied EvoProDom to the collection of organisms (EvoProDomDB) and found a total of 2042 mobile domains. Of these, 260 had undergone translocation events and 1782 were involved in indel events (Tables S1 and S3). Translocation events and indel event frequencies were grouped by Pfam super‐family [7, 8] (Table 2 and Table S5, respectively). Among the 10 most frequent domain super‐families were SH3 (Src homology‐3 domain), Ig (Immunoglobulin super‐family) and Ank (Table 2). The most frequent super‐families of mobile domains involved in indel events were ‘Unknown’, P‐loop_NTPase and TPR (Table S5).

Translocation events observed in the SH3 super‐family members were as follows: SH3_2 (239 translocations), SH3_1 (198 translocations), and SH3_9 (193 translocations). SH3 (src Homology‐3) domains are small protein domains approximately 50 amino acids in length [39, 40] and are found in various membrane‐associated or intracellular proteins [41, 42, 43], such as fodrin and yeast actin‐binding protein (ABP‐1). Additionally, SH3 domains mediate PPIs by facilitating protein complex assembly [37]. Translocation events observed in the Ig super‐family were as follows: Ig_3 (533 translocations), ig (219 translocations), I‐set (135 translocations), V‐set (117 translocations) and Ig_2 (116 translocations), C2‐set_2 (23 translocations), Ig_6 (5 translocations), and C1‐set (1 translocation). These domains are found in cell surface proteins and in intracellular muscle proteins (I‐set) and in the vertebrate immune system (V‐set) [44, 45]. The Ank repeats super‐family comprises Ank_2 (231 translocations), Ank_4 (184 translocations), Ank_5 (94 translocations), Ank_2 (19 translocations), and Ank_3 (1 translocation). These repeats are involved in PPIs that regulate cell cycle transition from G1 to S [33, 34, 35]. Such regulation is achieved by inhibitors of cyclin‐dependent kinase 4 protein complex formation and inhibition of CDK4/6 proteins [35]. These findings reveal that protein domains enriched in chimeric transcripts underwent many translocations. This supports a connection between chimeric transcripts and EvoProDom translocations. In addition, translocation events for protein domains, such as P kinase and ubiquitin, are found in multiple events and formed new fusions. Moreover, one domain encoded in each novel transcript underwent a translocation event [5]. Note that super‐families with the most and least number of translocations, SH3 (630) and SH2‐like (6), were enriched in chimeric transcripts (Table 2).

## Discussion

Here, we presented a novel protein evolution model, EvoProDom, which was based on the ‘mix and merge’ of protein domains. The EvoProDom model was implemented with and tested on EvoProDomDB, which consists of genomic and proteome data, along with orthologous protein and protein domain data, from 109 organisms from diverse taxa. In the EvoProDom model, translocations, and indel and duplication events were defined to reflect changes in domain content of a protein in orthologous groups. Moreover, in this model, such changes in protein domain composition were manifested at the organism level. Thus, SH3, which binds ligands [37, 38] and mediates PPIs [46], was observed as a highly abundant protein domain in translocations. Repetitive domains, such as Ank [33, 34, 35] and WD40 [36], appeared in multiple copies in proteins. Generally, 3D confirmations mediate PPIs [33, 34, 35, 36] by modulating protein networks of parent proteins. This modulation is mediated by novel PPIs of chimeric proteins [47]. Indeed, such domains, for example, SH3_2, Ig, and Ank_2 and others (see Results), were enriched in multiple fusion event‐generated chimeric transcripts [5]. As hypothesized, these domains participated in a high number of evolutionary translocation events. A probable explanation for the high frequency of these translocation events is the repetition of these domains. In general, fusions are produced by slippage of two parent genes. Fusion genes lose domains at junction sites. As a result, the proper function of the chimeric protein is impaired [47]. For example, fusion within the catalytic domain would render the protein nonfunctional. Selection would thus be against such a fusion. Repetitive domains, which appear in high copy numbers, would appear in chimeras at higher frequencies than expected from their sheer numbers alone, albeit due to selection, with lower repeats. Indeed, their average copy numbers were reduced in chimeric transcripts [5]. In EvoProDom, abundant domains or repetitive domains, for example, SH3, within KO groups, resulted in higher numbers of distinct DAs. This translates into a higher number of (ko, item) pairs (see Materials and methods). Consequently, these domains contribute more to the pool of mobile domains from which translocation events were generated, and were thus highly abundant in translocation events. Collectively, these results indicate that translocation events involving repetitive domains and highly abundant domains rewire PPI networks to achieve adaptive evolution.

The introduction of new organisms into EvoProDomDB required only full genomes and annotated proteomes. Orthologous protein and protein domain content data were identified from protein sequences using KoFamKOALA [6] and the Pfam search tool [7, 8]. Therefore, usage of these tools enables the extension of EvoProDom to any new organism with a full genome and annotated proteome. Moreover, the combined use of these tools provides a general method for obtaining protein domain content and orthologous protein annotation from protein sequence. In conclusion, EvoProDom presents a novel model for protein evolution based on the ‘mix and merge’ view of protein domains rather than DNA‐based models. This confers the advantage of considering chromosomal alterations in evolutionary events.

## Conflict of interest

The authors declare no conflict of interest.

## Author contributions

MFM designed, supervised the study and wrote the paper; GC and AG produced the data, verified results, and wrote the paper.

## Supporting information

**Table S1**. EvoProdom translocations.
**Table S2**. Superdomain translocations counts based on mobile domain.
**Table S3**. Raw indel events.
**Table S4**. Indel frequencies for indel classes based on mobile domain.
**Table S5**. Indel events per superfamily (counts).

## Data Availability

All data and methods are contained within the paper.
