# Supplementary material for: EvoProDom: evolutionary modeling of protein families by assessing translocations of protein domains
Source: FEBS Open Bio. 2021 Aug 21;11(9):2507–24. doi: 10.1002/2211-5463.13245 (PMC8409312; doi:10.1002/2211-5463.13245)
Supplement: Supplementary file 2 [file FEB4-11-2507-s001.pdf]

Supplementary materials

EvoProDom: Evolutionary modeling of protein families by assessing translocations of protein domains

Gon Carmi[1,†], Alessandro Gorohovski [1,†], Milana Frenkel-Morgenstern [1,*]

[1]Cancer Genomics and BioComputing of Complex Diseases Lab, The Azrieli Faculty of Medicine, Bar-Ilan University, 8 Henrietta Szold St, Safed 13195, ISRAEL

*Corresponding Author:

e-mail: milana.morgenstern@biu.ac.il

[†] These authors contributed equally to this work.

Supplementary tables

Table S1 EvoProdom translocations. Translocations are characterized by mobile domains, orthologous groups (KO, A1, B1, A2, B2) and organisms (A*_orgs, B*_orgs) classified based on superdomain taxonomy*. These organism groups are assigned a representative superdomain taxonomy if all organisms share same supderdomain taxonomy. Otherwise, they are assigned as "Mixed" (A*_orgs_class, B*_orgs_class). Finally, translocations are classified based on the organism group classification into superdomains (A*_orgs class-B*_orgs_class), e.g., Eukaryota-Eukaryota, which represents a majority of translocations (over 99%). This is due to overrepresentation of Eukaryota species. *Superdomain taxa are Eukaryota,Viruses and Bacteria. Superfamily (clan) information is provided.

Table S2 Superdomain translocations counts based on mobile domain. Translocations are characterized by mobile domains in organisms classified based on superdomain taxonomy*. These organism group are assigned a representative superdomain taxonomy if all organisms share same supderdomain taxonomy. Otherwise, they are assigned as "Mixed". Finally, translocations are classified based on organism group classification into superdomains, e.g., Eukaryota-Eukaryota, which represents a majority of translocations (over 99%). The most frequent domain for Eukaryota-Eukaryota is Ig_3. Related to Table S1. *Superdomain taxa are Eukaryota, Viruses and Bacteria. Superfamily (clan) information is provided.

Table S3 Raw indel events. Indel events are composed of a domain, an orthologous protein group (KO) and two groups of organisms, such that the domain is missing from a protein group for a group of organisms (missing organisms) and is gained for the other organism group (gained organisms). Additionally, indels are classified to groups, denoted as missing superdomain-gained superdomain, based on superdomain taxonomy* of missing and gained organisms or Mixed_missing and Mixed_gained**, respectively. For example, Eukaryota-Eukaryota signify that the domain is missing and gained between eukaryotes and accounts for over 96% of indel events. *Superdomain taxa are Eukaryota, Viruses and Bacteria. **Organism groups are assigned representative superdomain taxonomy if all organisms share same supderdomain taxonomy. Otherwise, they are assigned as "Mixed". Mixed_missing is denoted as Mixed-any and Mixed gained as any-Mixed. Superfamily (clan) information is provided.

Table S4 Indel frequencies for indel classes based on mobile domain. The most frequent domain for indel class Eukaryota-Eukaryota is SNF2_N, with 290 indel events which belong to P-loop_NTPase superfamily (clan). Superfamily name (clan) is included. Related to Table S3.

Table S5 Indel events per superfamily (counts). Superfamily annotation is provided. Indel frequencies for indel classes based on superfamily (clan).
The most frequent clan for indel class Eukaryota-Eukaryota is "Unknown" with the 8382 indel. Superfamily name (clan) is included Related to Table S3.
